# Eco-Friendly Extraction and Formulation of Black Sea Shark Liver Oil-Based Emulgel for Anti-Inflammatory and Healing Dermatocosmetic Applications

**DOI:** 10.3390/gels11040222

**Published:** 2025-03-21

**Authors:** Sorinel Marius Neacșu, Lucian Hîncu, Lavinia Lia Vlaia, Dumitru Lupuliasa, Alexandru Scafa-Udriște, Sebastian Mihai, Gabriel Olteanu, Alexandru Mihai Grumezescu, Răzvan Ene, Ruxandra Cristina Marin, Magdalena Mititelu

**Affiliations:** 1 Department of Pharmaceutical Technology and Biopharmacy, Faculty of Pharmacy, “Carol Davila” University of Medicine and Pharmacy, 6 Traian Vuia Street, 020945 Bucharest, Romania; sorinel-marius.neacsu@drd.umfcd.ro; 2 Department of Drug Industry and Pharmaceutical Biotechnologies, Faculty of Pharmacy, University of Medicine and Pharmacy Carol Davila, 020956 Bucharest, Romania; lucian.hincu@umfcd.ro; 3 Department of Pharmaceutical Technology, Formulation and Technology of Drug Research Center, “Victor Babeș” University of Medicine and Pharmacy, 300041 Timișoara, Romania; vlaia.lavinia@umft.ro; 4 Department of Cardio-Thoracic Pathology, Faculty of Medicine, “Carol Davila” University of Medicine and Pharmacy, 050474 Bucharest, Romania; 5 Department of Therapeutic Chemistry, Faculty of Pharmacy, “Ovidius“ University of Constanta, 6 Căpitan Aviator Al Șerbănescu Street, 900470 Constanta, Romania; sebastian.mihai@univ-ovidius.ro; 6 Department of Clinical Laboratory and Food Safety, Faculty of Pharmacy, “Carol Davila” University of Medicine and Pharmacy, 6 Traian Vuia Street, 020945 Bucharest, Romania; gabriel.olteanu@mst.umfcd.ro (G.O.); magdalena.mititelu@umfcd.ro (M.M.); 7 Department of Science and Engineering of Oxide Materials and Nanomaterials, National University of Science and Technology Politehnica Bucharest, 011061 Bucharest, Romania; agrumezescu@upb.ro; 8 ICUB—Research Institute of The University of Bucharest, University of Bucharest, 011061 Bucharest, Romania; 9 Clinical Department No. 14, Faculty of Medicine, “Carol Davila” University of Medicine and Pharmacy, 050474 Bucharest, Romania; razvan.ene@umfcd.ro; 10 Department of Pharmacology, Clinical Pharmacology and Pharmacotherapy Faculty of Medicine, “Carol Davila” University of Medicine and Pharmacy, 050474 Bucharest, Romania; ruxandra.marin@umfcd.ro

**Keywords:** shark liver oil, borage oil, emulgel formulation, anti-inflammatory activity, wound healing, dermatocosmetic applications, omega-3 fatty acids, rheological properties

## Abstract

This study explores the eco-friendly extraction and formulation of emulgels based on Black Sea shark liver oil for their potential anti-inflammatory and wound healing dermatocosmetic applications. Two emulgel formulations were prepared: Gel 1, containing shark liver oil, and Gel 2, combining shark liver oil with borage oil. The eco-friendly extraction of shark liver oil was performed, yielding a high content of polyunsaturated omega-3 fatty acids, primarily eicosapentaenoic acid (16.68 ± 0.28 mg/g %) and docosahexaenoic acid (18.14 ± 0.31 mg/g %). Physicochemical evaluations of the emulgels revealed excellent stability over time, with small variations in pH, viscosity, and spreadability, confirming their robustness. Rheological analysis demonstrated pseudoplastic behavior for both formulations, with Gel 2 exhibiting a more favorable flow and consistency index (K = 34.11, n = 0.28) compared to Gel 1 (K = 32.73, n = 0.29). The anti-inflammatory effect was evaluated using two experimental edema models: 10% kaolin suspension and 6% dextran solution. Both emulgels demonstrated significant edema reduction, with Gel 2 showing a more potent anti-inflammatory effect. The wound healing effect was assessed in vivo, revealing that Gel 2 accelerated wound closure and hair restoration, outperforming Gel 1 and the reference diclofenac gel. These results suggest that Black Sea shark liver oil-based emulgels, especially borage oil formulation, offer promising eco-friendly alternatives for dermatocosmetic applications, with enhanced anti-inflammatory and wound healing properties.

## 1. Introduction

The marine environment represents one of Earth’s most essential natural reservoirs, providing biologically active foods, such as fish and seafood, with significant nutritional and therapeutic potential. Marine biodiversity is important in supporting human health and disease prevention and management while promoting overall physiological functions. Seafood, particularly fish, is recognized for its valuable lipid profile, characterized by a low content of saturated fatty acids and a high concentration of polyunsaturated fatty acids (PUFAs), notably omega-3 fatty acids, which are essential for the cardiovascular system, cognitive function, and inflammation modulation [1]. Fish serve as a rich source of amino acids with high biological value, including lysine and methionine, as well as proteins with high content of essential amino acids. Fatty fish such as herring, mackerel, trout, anchovies, salmon, and sardines, along with oils derived from cod and tuna liver, are particularly rich in eicosapentaenoic acid (EPA) and docosahexaenoic acid (DHA), well-documented for their cardioprotective and anti-inflammatory properties [1,2,3,4]. Additionally, marine-derived foods contain sterols that reduce low-density lipoprotein (LDL) cholesterol levels, further supporting cardiovascular health [5]. Fish oils are excellent sources of fat-soluble vitamins, particularly vitamins A, D, and E. Cod and halibut liver oils, for instance, are rich in vitamins A and D, critical for immune function and bone mineralization. Other species, including herring, mackerel, salmon, and freshwater trout, also contain significant amounts of vitamin D, essential for bone metabolism and preventing deficiencies such as osteomalacia and rickets [6,7].

Beyond their vitamin content, marine-derived foods are abundant in essential minerals and trace elements, often present in higher concentrations than in terrestrial sources. Calcium, the most prevalent mineral in fish, is fundamental for maintaining bone integrity. Other essential micronutrients found in seafood, including copper, selenium, zinc, and magnesium, contribute to physiological homeostasis and metabolic regulation [8,9,10,11,12,13,14].

This exceptional nutritional profile highlights the role of fish as a functional food with broad health benefits, reinforcing its importance in balanced dietary patterns and disease prevention strategies.

Shark liver oil (SLO) supplementation has begun to be widely used in the prophylaxis and treatment of certain pathologies of the human body. Studies have demonstrated the beneficial effect of fish oil treatment in lipid metabolism disorders, pulmonary inflammation, digestive tract diseases, lymphadenopathy, cancer, dermatitis, and wound healing [15,16,17].

SLO is purified from shark species such as *Centrophorus squamosus*, *Cetorhinus maximus*, *Squalus acanthias*, basking sharks, and dogfish sharks that live in cold and deep oceans. SLO is rich in alkylglycerols (AKGs), pristane, squalene, vitamin A, vitamin D, fatty acid esters, triglycerides, cholesterol, and omega-3 polyunsaturated fatty acids, and these bioactive compounds seem to be involved in the decrease in the incidence of cancer in sharks in general [18]. Scandinavian fishermen use this oil with amazing therapeutic properties to treat certain pathologies, such as wounds, infertility, tumors, and even cardiovascular diseases [15]. The composition of shark liver oil differs between shark species and depends on the shark’s size since the liver represents approximately 25% of the total weight of the shark [18,19]. In the *Centrophorus* species, it was found that 60% to 90% of the weight of the liver is due to oil rich in squalene, the concentration of which increases with the shark’s age. At the same time, more than 50 fatty acids have been identified [20].

Squalene, a triterpene first isolated from shark liver oil (*Squalus* sp.), is one of its richest natural sources [21]. As a precursor of steroid hormones and cholesterol, squalene exhibits potent antioxidant properties, protecting cells from oxidative stress and lipid peroxidation [22,23]. Widely used in dermatological products, it is an emollient and moisturizer due to its affinity for the skin’s lipid barrier [24]. In shark liver, squalene constitutes 40–60% of the liver mass, with species like *Centrophorus moluccensis* containing up to 95% squalene in their liver oil [25]. Beyond skincare, squalene demonstrates anti-inflammatory properties, modulating prostaglandin E2 (PGE2) levels and reducing COX-2 expression in tumor tissue. A study on guinea pigs with transplanted tumors treated with doxorubicin (DOX) found that squalene significantly lowered PGE2 levels (*p* < 0.05), suggesting its potential as an adjuvant in chemotherapy [26].

Omega-3 polyunsaturated fatty acids play an essential role in protecting the cardiovascular system, counteracting the adverse effects of Western dietary patterns associated with increased cardiovascular disease (CVD) risk [27,28,29,30,31,32,33,34,35,36].

SLO has been researched and successfully used as an adjuvant in the treatment regimen of oncological patients (activates macrophages, inhibits angiogenesis at the tumor level, reduces inflammation caused by chemotherapeutic agents), in strengthening the immune system, reducing the incidence of cardiovascular events (atherosclerosis, myocardial infarction, hypercholesterolemia, hyperlipidemia), improving fertility, improving skin health and elasticity, reducing mouth ulcers, and preventing tissue damage induced by radiotherapy [37,38,39,40,41,42,43,44,45,46,47,48,49].

SLO has garnered attention for its therapeutic potential in managing various dermatological diseases thanks to its rich composition of bioactive compounds, including AKG, squalene, and polyunsaturated omega-3 fatty acids. Clinical studies have highlighted its efficacy in treating chronic inflammatory skin conditions such as eczema, psoriasis, and acne and promoting wound healing in chronic ulcers and burns [50,51]. Squalene, a potent antioxidant, protects the skin from oxidative stress and environmental damage, improving skin hydration and elasticity and making it beneficial in conditions where the skin barrier is compromised, like eczema and psoriasis. AKG, another key constituent, has been shown to activate macrophages, enhance local immune response, promote tissue regeneration, accelerate wound healing, and reduce the risk of infection in chronic ulcers or burns [52,53]. The anti-inflammatory effects of omega-3 fatty acids, particularly EPA and DHA, play a critical role in modulating immune responses by downregulating the production of pro-inflammatory cytokines such as TNF-α and IL-6. This is especially important in inflammatory skin disorders like psoriasis, where the overproduction of these cytokines contributes to the rapid turnover of skin cells. Overall, SLO provides a multifaceted mechanism of action that supports skin regeneration, reduces inflammation, and improves the skin’s ability to repair and protect itself, making it a valuable adjunct in treating various dermatological diseases [54,55].

Marine pollution poses a significant challenge to the quality and safety of marine-derived nutraceuticals, including fish and shark liver oils [56]. Contaminants such as heavy metals (mercury, lead, cadmium), persistent organic pollutants (POPs), and microplastics can accumulate in marine organisms, potentially affecting the purity and bioavailability of essential nutrients like omega-3 fatty acids. To mitigate these risks, advanced purification techniques are employed to remove contaminants while preserving the nutritional value of marine oils. Sustainable sourcing and regular quality control measures are essential to ensure marine nutraceuticals meet safety standards [57,58]. Increasing awareness and regulatory efforts are essential for maintaining the health benefits of marine-derived supplements while addressing environmental concerns related to ocean pollution.

This study presents an original approach to harnessing the therapeutic potential of Black Sea shark liver oil (*Squalus acanthias*) by focusing on its unique composition of bioactive fatty acids. The paper aims to analyze the characteristics of Black Sea shark liver oil by highlighting the main categories of fatty acids in its composition in order to capitalize on the therapeutic potential by formulating dermatocosmetic preparations with anti-inflammatory and healing potential. In this regard, an eco-friendly extraction method was used to protect the bioactive principles of liver oil collected from Black Sea shark specimens (*Squalus acanthias*). Two original emulgel formulas were developed and tested on laboratory animals to evaluate their healing and anti-inflammatory actions.

## 2. Results and Discussion

### 2.1. Characteristics of the Therapeutic Oils Analyzed

The analyzed physicochemical parameters are presented in Table 1. A higher iodine value was observed for shark liver oil, indicating a greater content of unsaturated fatty acids compared to borage oil.

The fatty acid composition of shark liver oil and borage oil highlights distinct nutritional profiles and potential health implications (Table 2). Shark liver oil is rich in polyunsaturated fatty acids (PUFAs) from the omega-3 family, particularly eicosapentaenoic acid (EPA) (16.68 ± 0.28 mg/g) and docosahexaenoic acid (DHA) (18.14 ± 0.31 mg/g).

Conversely, borage oil is predominantly composed of omega-6 PUFAs, with high levels of linoleic acid (29.86 ± 0.16 mg/g) and gamma-linolenic acid (GLA) (26.66 ± 0.37 mg/g).

**Figure 1:**
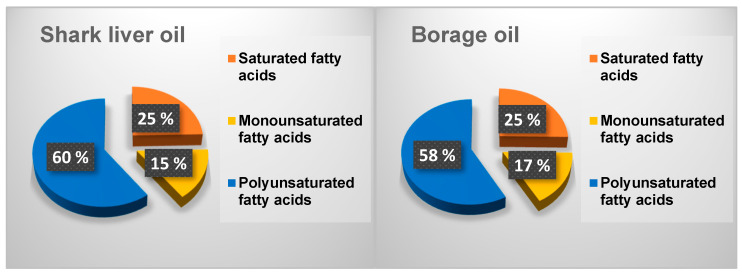
Distribution of different fractions of fatty acids in the oil samples.

The differences in the fatty acid distribution between shark liver oil and borage oil, as illustrated in Figure 1, highlight key compositional variations that influence their functional and nutritional properties. While both oils contain a mixture of saturated (SFAs), monounsaturated (MUFAs), and polyunsaturated fatty acids (PUFAs), their relative proportions contribute to distinct biochemical and physiological effects.

Borage oil exhibits a slightly higher saturated fatty acid content than shark liver oil (Figure 1). Saturated fatty acids play a role in structural stability within cell membranes and serve as an energy source, but excessive intake has been associated with increased cardiovascular risk. The presence of a higher monounsaturated fatty acid fraction in borage oil, primarily oleic acid, is beneficial for maintaining membrane fluidity and supporting cardiovascular health by improving lipid profiles and reducing inflammation. Oleic acid is also known for enhancing skin barrier function, making borage oil a valuable ingredient in dermatological applications [59].

On the other hand, shark liver oil contains a higher proportion of polyunsaturated fatty acids, particularly from the omega-3 category, including EPA and DHA. These long-chain PUFAs are well-recognized for their anti-inflammatory and cardioprotective properties, which regulate immune responses and neurological function. The higher PUFA content in shark liver oil also suggests greater oxidative susceptibility, making storing and formulating the oil with appropriate antioxidants essential to maintaining stability [60].

The observed differences in fatty acid distribution suggest that each oil has unique functional applications. Borage oil, with its balance of SFAs, MUFAs, and omega-6 PUFAs, is well-suited for applications in skincare, inflammatory conditions, and metabolic health. In contrast, enriched in omega-3 PUFAs, shark liver oil is better positioned for cardiovascular and cognitive health support [61]. The complementary nature of these oils suggests potential benefits in combining them for synergistic effects, optimizing their fatty acid profiles for improved therapeutic applications.

### 2.2. Emulgels Characteristics

The selection of a 2% Carbopol gel base to develop emulgels was driven by its proven efficacy in forming a stable, homogeneous, and bioadhesive matrix suitable for topical application. Carbopol ensures optimal viscosity, facilitating controlled drug release, enhanced skin penetration, and prolonged contact time with the application site. Its ability to form a hydrogel network provides a non-greasy, smooth texture, improving patient compliance. The total emulsified oil phase of 5% was chosen to balance incorporating bioactive lipid components while maintaining stability and spreadability. This concentration allows for effective emulsification without phase separation, ensuring consistent delivery of shark liver oil and borage oil’s therapeutic benefits while preserving the mechanical and rheological properties essential for an ideal topical formulation [62].

The combination of shark liver oil and borage oil in the second emulgel formula (Gel 2) is expected to offer enhanced therapeutic effects due to their bioactive components’ complementary and synergistic properties. Shark liver oil is characterized by a high content of omega-3 PUFAs, particularly EPA and DHA, as well as bioactive alkylglycerols. EPA and DPA are known for their potent anti-inflammatory, immunomodulatory, and skin-repair properties. These omega-3 fatty acids inhibit pro-inflammatory mediators such as prostaglandins and leukotrienes, reducing local inflammation and accelerating wound-healing. Additionally, alkylglycerols contribute to immune support, antimicrobial activity, and cell membrane integrity, which may improve tissue regeneration and protect against microbial contamination in damaged skin [63,64].

Borage oil, on the other hand, is rich in omega-6 PUFAs, notably linoleic acid and GLA. Linoleic acid is important in maintaining skin barrier function, improving hydration, and enhancing epidermal integrity. It is an essential fatty acid for synthesizing ceramides, which helps restore and protect the skin’s natural moisture barrier. GLA, a unique component of borage oil, is metabolized into dihomo-gamma-linolenic acid (DGLA), a precursor to anti-inflammatory prostaglandins (PGE1). This pathway helps mitigate inflammation and can be particularly beneficial in treating skin disorders characterized by dryness, irritation, or compromised barrier function, such as eczema and atopic dermatitis [65,66].

The combination of shark liver oil and borage oil in Gel 2 is expected to deliver a balanced omega-3/omega-6 fatty acid profile, addressing both inflammatory and structural skin needs. While omega-3 fatty acids reduce acute inflammation and promote tissue repair, omega-6 fatty acids restore hydration, improve elasticity, and protect the skin from further damage. This dual-action formulation may offer superior wound-healing properties by reducing inflammation, promoting cellular repair, and improving moisture retention. Additionally, including both oils supports the long-term maintenance of skin health, as their lipid composition mimics the natural structure of the epidermis, enhancing dermal absorption and bioavailability.

Furthermore, this combination may enhance patient compliance by providing a non-greasy, easily absorbed formulation with prolonged effects on skin regeneration and hydration.

The stability assessment of the prepared emulgels, as presented in Table 3, confirms that both formulations—Gel 1 (shark liver oil emulgel) and Gel 2 (shark liver oil and borage oil emulgel)—maintained their physicochemical properties over time, with only minor variations observed. The pH measurements remained within an acceptable range for topical application (4.7–5.5), suggesting that the formulations are compatible with the skin’s natural pH, thereby minimizing the risk of irritation or discomfort.

The recorded values showed only slight fluctuations in viscosity, indicating that the emulgel matrix retained its structural integrity and consistency over time. This is essential for ensuring ease of application, adherence to the skin, and sustained release of bioactive compounds. The carbopol-based gel structure, known for its good rheological properties and stability, likely contributed to maintaining the desired consistency and preventing phase separation or syneresis.

Additionally, the macroscopic appearance of the formulations showed no significant changes, suggesting that the emulsification process effectively achieved a homogeneous and stable emulsion system. The absence of visible oil phase separation or significant color/texture alterations over time further supports the long-term stability of these formulations, which is essential for ensuring their practical usability and consumer acceptability.

These results, together with SEM analysis, reinforce that the emulsified oil phase (5%) and the 2% carbopol gel base provided an adequate formulation balance, ensuring a stable and effective delivery system for the bioactive components of shark liver oil and borage oil. The slight differences between the two formulations, particularly in viscosity and droplet distribution, may influence their application properties and release profiles. Still, both demonstrate sufficient robustness for extended storage and clinical use.

Scanning Electron Microscopy (SEM) analysis was performed to examine the morphological characteristics and internal microstructure of the two emulgel formulations, Gel 1 (shark liver oil emulgel) and Gel 2 (shark liver oil and borage oil emulgel).

**Figure 2:**
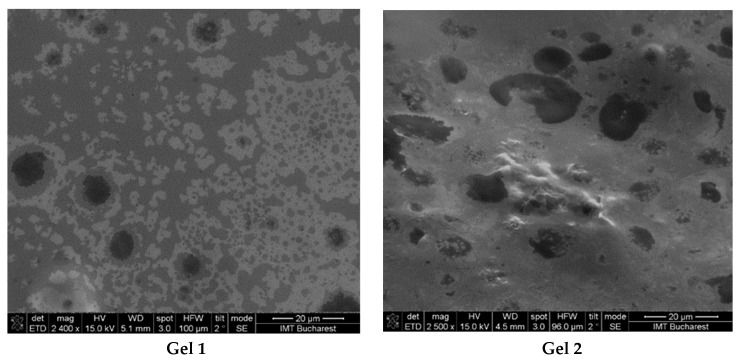
SEM images from emulgels: Gel 1—emulgel with shark liver oil; Gel 2—emulgel with shark liver oil and borage oil.

The SEM images revealed significant differences in the structural organization, droplet distribution, and surface homogeneity of the two formulations (Figure 2).

The micrographs of Gel 1 showed a uniform and well-organized network, indicative of a stable emulgel structure. The oil droplets appeared finely dispersed within the polymeric carbopol matrix, suggesting effective emulsification and stabilization. The microstructure exhibited a smooth and uniform texture, which may enhance viscoelastic properties and support prolonged skin retention. The small and homogeneously distributed oil droplets suggest good phase compatibility, essential for the controlled release of bioactive compounds from shark liver oil.

For Gel 2, SEM analysis revealed a slightly different internal structure due to the incorporation of borage oil, which affected the droplet size and distribution. Compared to Gel 1, the incorporation of omega-6-rich borage oil resulted in a more heterogeneous oil droplet distribution characterized by increased droplet size and irregular morphology.

Both formulations demonstrated stable emulsion systems with well-defined microstructures, confirming successful emulsification and gel formation. The SEM findings aligned with the expected rheological and functional properties, indicating that both formulations were structurally robust and suitable for topical application.

**Figure 3:**
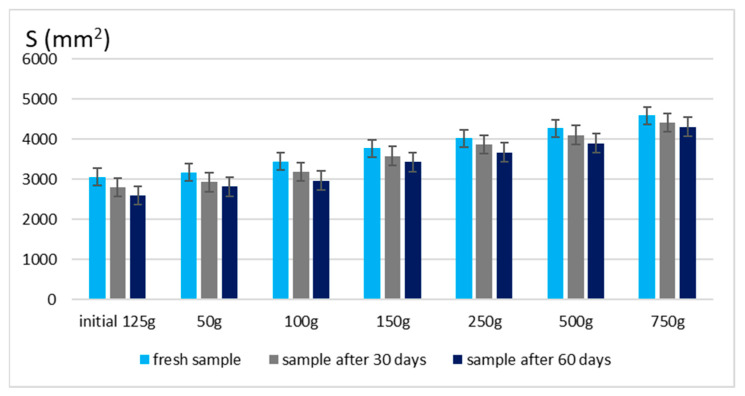
Spreading area for emulgel with shark liver oil.

**Figure 4:**
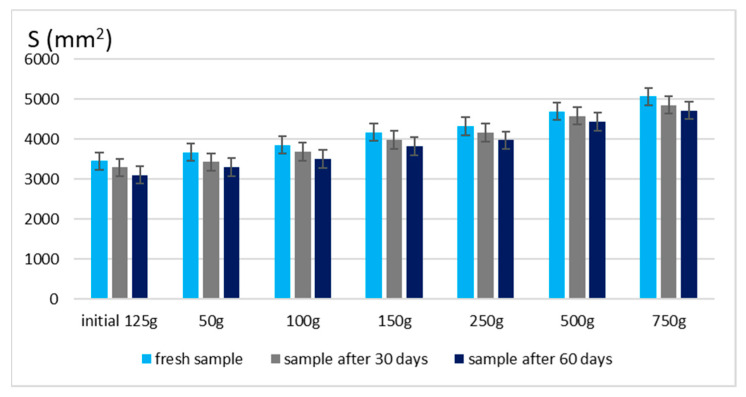
Spreading area for emulgel with shark liver oil and borage oil.

The spreadability of a topical formulation is a key parameter that influences ease of application, uniform distribution, and overall user experience. Based on the results presented in Figure 3 and Figure 4, the spreadability values for both Gel 1 (shark liver oil emulgel) and Gel 2 (shark liver oil and borage oil emulgel) remained within an optimal range over time, indicating that the formulations retained their desirable application properties.

Maintaining good spreadability over time ensures easy application and effective bioactive compound delivery. A well-spread formulation enhances skin coverage and penetration, allowing for efficient absorption of the active ingredients. The results confirm that the developed emulgels possess desirable rheological properties, ensuring ease of use, optimal skin adhesion, and sustained therapeutic benefits without excessive stickiness or greasiness.

The rheological evaluation of the two emulgels provides valuable insights into their flow behavior and stability over time (Table 4 and Table 5). Both formulations exhibited pseudoplastic behavior, as reflected in the flow index values (n < 1), suggesting shear-thinning properties beneficial for topical applications, as they allow for easier spreading under mechanical stress. The yield stress values (τ_0_) were slightly higher for Gel 2 (52.366 Pa) compared to Gel 1 (46.233 Pa), indicating that the combination of shark liver oil and borage oil may contribute to a more structured gel network, potentially enhancing the stability and controlled release of active compounds. Regarding viscosity, both formulations maintained consistency over the 30 days, with only slight decreases observed at a low shear rate of 0.3 rpm. Gel 1 exhibited a viscosity decrease from 882.700 Pa·s to 876.200 Pa·s, while Gel 2 decreased from 844.600 Pa·s to 832.600 Pa·s, demonstrating good long-term stability. The consistency index (K) values further support these observations, with Gel 2 presenting a slightly higher value (34.114 Pa·s^n^) than Gel 1 (32.738 Pa·s^n^), which may be attributed to the additional structural complexity provided by borage oil components (Table 4).

The Herschel–Bulkley model yielded high correlation coefficients (0.988 for Gel 1 and 0.992 for Gel 2), indicating that this model accurately represents the emulgels’ non-Newtonian flow behavior (Table 5).

**Figure 5:**
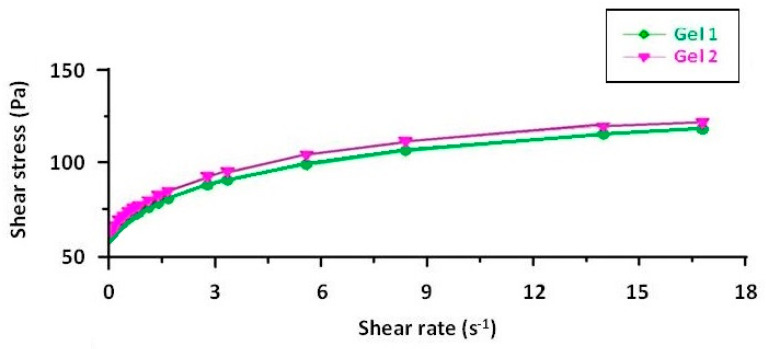
Rheograms of emulgels: Gel 1—emulgel with shark liver oil; Gel 2—emulgel with shark liver oil and borage oil.

**Figure 6:**
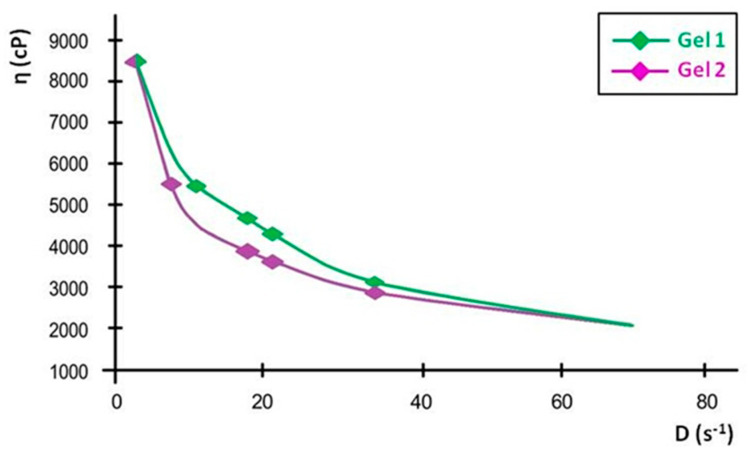
Flow curves of emulgels: Gel 1—emulgel with shark liver oil; Gel 2—emulgel with shark liver oil and borage oil.

The rheograms for both emulgels, Gel 1 and Gel 2, illustrate their non-Newtonian, shear-thinning behavior, confirming their suitability for topical application (Figure 5). The flow curves plot shear stress against shear rate and exhibit a non-linear increase, characteristic of pseudoplastic fluids (Figure 6). Both formulations require an initial yield stress to initiate flow, as seen in the Herschel–Bulkley model parameters, with Gel 2 showing a slightly higher yield stress than Gel 1. This suggests that Gel 2 has a slightly stronger internal structure, likely due to the combined effects of shark liver oil and borage oil.

The downward curvature of the flow curves at increasing shear rates indicates that both emulgels exhibit shear-thinning behavior, meaning their viscosity decreases as the shear rate increases. This property is highly advantageous for topical formulations, as it ensures ease of spreading under manual application while maintaining sufficient viscosity at rest to prevent runoff. The consistency index values further support this observation, showing a slight difference between the two formulations, reinforcing that Gel 2 maintains a slightly more structured network.

The viscosity curves, plotted as viscosity versus shear rate, show a progressive decline in viscosity with increasing shear rate, confirming the shear-thinning properties. At the lowest measured shear rate, Gel 1 had an initial viscosity that remained stable over time, while Gel 2 initially had a slightly lower viscosity, with a minor decrease over time. These small variations indicate that both formulations maintain their structural integrity over time.

**Figure 7:**
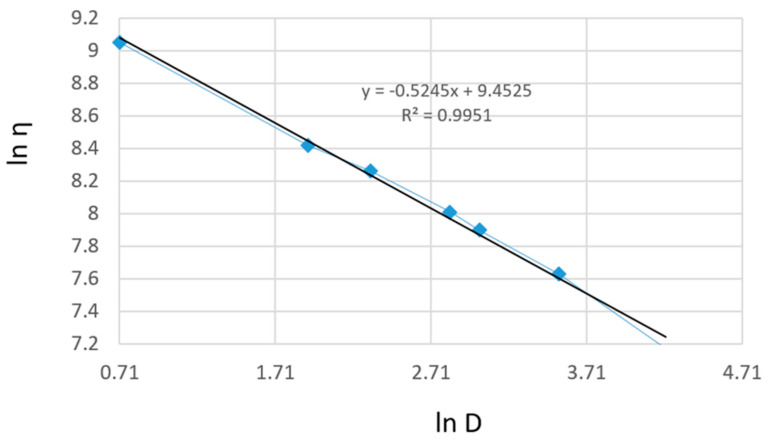
Linearization of rheological parameters with respect to the power law for shark liver oil emulgel: experimental data line (blue); power law linear fit line (black).

The linearization of rheological parameters for the shark liver oil emulgel, as shown in Figure 7, demonstrates the applicability of the power law model in describing its flow behavior. The obtained parameters suggest that the emulgel exhibits non-Newtonian, shear-thinning properties, where the viscosity decreases with increasing shear rate.

The linear relationship observed in Figure 7 confirms that the experimental data fit well within the Ostwald–de Waele model, with high correlation coefficients supporting the model’s accuracy [67]. This behavior is advantageous for topical formulations, ensuring easy application while maintaining structural integrity at rest. The consistency index and flow index values indicate a stable formulation with good spreadability, which is essential for ensuring uniform coverage on the skin.

The rheological characterization of shark liver oil using the Ostwald–de Waele model provides valuable insights into its flow behavior (Table 6). The consistency index (K) values obtained from the three tests indicate a relatively stable viscosity profile, with an average value of 9.43. The low coefficient of variation (3.21%) further confirms the consistency of these measurements, suggesting minimal variability in the oil’s rheological properties.

The flow index (n) values range between 0.443 and 0.501, with an average of 0.473, indicating that shark liver oil exhibits shear-thinning behavior, characteristic of non-Newtonian fluids. This suggests that its viscosity decreases under shear stress, which is a favorable property for topical formulations, as it allows for ease of application and spreading while maintaining structural integrity at rest. The higher coefficient of variation for the flow index (6.14%) suggests slightly more variability in this parameter, which could be attributed to natural compositional variations in the oil.

The correlation coefficient (R) values, all above 0.99, confirm an excellent fit of the experimental data to the Ostwald–de Waele model, validating its use in describing the flow behavior of shark liver oil. These findings indicate that shark liver oil has suitable rheological properties for incorporation into semi-solid formulations such as emulgels, where controlled spreadability and stability are crucial for effective topical application.

**Figure 8:**
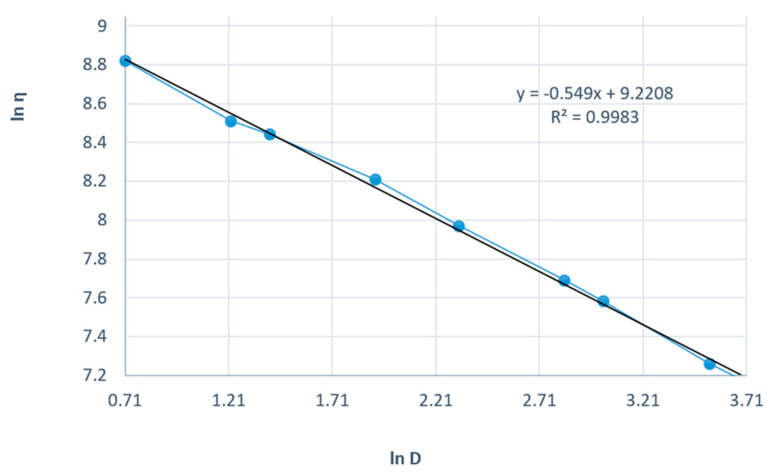
Linearization of rheological parameters with respect to the power law for shark liver oil and borage oil emulgel: experimental data line (blue); power law linear fit line (black).

Figure 8 presents the linearization of the rheological parameters according to the power law for the shark liver oil and borage oil emulgel. This graph plots the natural logarithm of shear stress (τ) against the natural logarithm of shear rate ($\dot{\gamma }$) to obtain a linear relationship, which allows the determination of the consistency index (K) and flow index (n) for the emulgel.

The linearity of the data, as shown by the high correlation coefficients, further supports the suitability of the power law model for describing the flow behavior of the emulgel. The slope of the resulting line corresponds to the flow index (n), which indicates the degree of shear thinning; values of n less than 1 signify that the formulation exhibits shear-thinning behavior, which is a characteristic desired in topical preparations to ensure ease of application and spreading on the skin.

The consistency index (K), derived from the intercept, measures the material’s viscosity at a given shear rate, reflecting its resistance to flow. The shark liver oil and borage oil emulgel results indicate moderate viscosity, indicating an appropriate balance between stability and spreadability for topical use.

The Ostwald–de Waele model parameters for the shark liver oil and borage oil emulgel indicate a non-Newtonian, shear-thinning behavior characterized by a flow index (n) lower than 1 (Table 7). The high correlation coefficient values (R ≈ 0.998) confirm that the experimental data fit well within the power law model, supporting its applicability in describing the flow properties of this formulation.

The consistency index (K) values are slightly lower compared to the shark liver oil-only emulgel, suggesting a minor reduction in viscosity, likely due to the presence of borage oil, which may influence the structural organization of the formulation. The flow index (n) variations indicate a moderate dependence of viscosity on shear rate, a desirable property for topical formulations, ensuring ease of application while maintaining consistency over time.

### 2.3. Evaluation of the Anti-Inflammatory Effect of Emulgels

**Figure 9:**
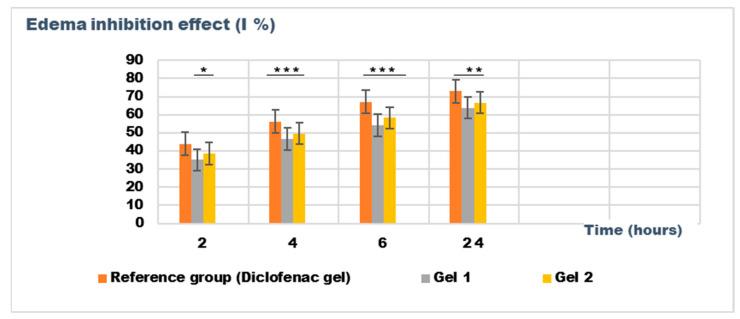
The evolution of the inhibition effect (I %) on inflammatory edema was induced by 10% kaolin suspension during the treatment. * *p* < 0.05, ** *p* < 0.01, and *** *p* < 0.001 refer to statistical significance between the reference group and tested group: Gel 1—emulgel with shark liver oil; Gel 2—emulgel with shark liver oil and borage oil.

**Figure 10:**
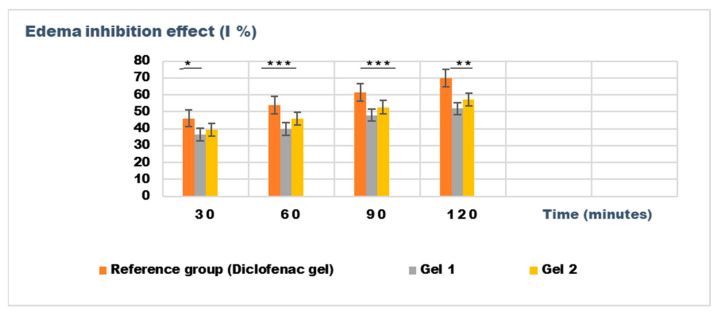
The evolution of the inhibition effect (I %) on inflammatory edema induced with dextran solution 6% during the treatment. * *p* < 0.05, ** *p* < 0.01, and *** *p* < 0.001 refer to statistical significance between the reference group and tested group: Gel 1—emulgel with shark liver oil; Gel 2—emulgel with shark liver oil and borage oil.

The experimental results of the evaluations of the anti-inflammatory effect of the tested emulgels are presented in Table 8 and Table 9. Figure 9 and Figure 10 show the evolution of the edema inhibition effect (I %) over time for the preparations used (emulgels Gel 1 and Gel 2, Diclofenac gel), compared to the untreated control group.

The experimental results from evaluating the anti-inflammatory effects of the tested emulgels (Gel 1 and Gel 2) on inflammatory edema induced by kaolin suspension and dextran solution provide valuable insights into their potential efficacy as anti-inflammatory agents.

The results of the kaolin-induced edema model indicate that both emulgels (Gel 1 and Gel 2) demonstrated significant reductions in edema compared to the control group. After 24 h, Gel 1 showed a marked decrease in edema (0.106 ± 0.025 mL) compared to the control group (0.293 ± 0.036 mL), with similar results observed for Gel 2 (0.098 ± 0.016 mL). Notably, the diclofenac gel also showed reduced edema (0.085 ± 0.046 mL), comparable to the emulgel formulations. The statistical analysis, however, revealed that the differences between the groups were not statistically significant (*p* > 0.05), indicating that all treatments, including the emulgels and diclofenac gel, had a similar level of effect on the reduction of kaolin-induced edema (Table 8).

The results from the dextran-induced edema model demonstrated a trend toward reduced edema in the treated groups, particularly at the 30 min and 60 min time points. At these intervals, both Gel 1 and Gel 2 exhibited reduced edema volume compared to the control group, with Gel 2 showing the most consistent results (e.g., 0.126 ± 0.014 mL at 30 min). The diclofenac gel showed an even greater reduction in edema, particularly at the 120 min mark (0.080 ± 0.044 mL), which aligns with its known anti-inflammatory effects. While the statistical analysis for dextran-induced edema (*p* > 0.05) also indicated no significant difference between groups, the trend observed across the different time points suggests that both emulgels may exhibit moderate anti-inflammatory potential (Table 9).

Both emulgels demonstrated a favorable trend in reducing inflammatory edema compared to the untreated control group. Gel 2 (a combination of shark liver oil and borage oil) performed slightly better than Gel 1 (shark liver oil emulgel). However, the lack of significant differences in the statistical analysis suggests that although the emulgels exhibited anti-inflammatory properties, they may not outperform the reference diclofenac gel regarding edema reduction under specific experimental conditions.

Figure 9 and Figure 10 illustrate the time-dependent evolution of the inhibitory effect on edema induced by inflammatory agents, confirming the efficacy of both tested emulgels in reducing inflammation. Notably, the results highlight a superior anti-inflammatory effect for Gel 2, which combines shark liver oil with borage oil, compared to Gel 1, which contains only shark liver oil. This suggests a synergistic interaction between the bioactive components of the two oils, leading to an enhanced suppression of edema formation.

Shark liver oil, known for its high content of squalene, alkylglycerols, and omega-3 fatty acids, exhibits well-documented anti-inflammatory and immunomodulatory properties, reducing inflammatory mediators and oxidative stress [68]. Meanwhile, borage oil, rich in gamma-linolenic acid (GLA), is a key precursor for prostaglandin E1 (PGE1) synthesis, which plays an important role in modulating inflammation and maintaining skin barrier function [69]. The combination of these bioactive lipids in Gel 2 likely enhances membrane stabilization, reduces vascular permeability, and modulates inflammatory cytokine production more effectively than shark liver oil alone.

Despite Gel 2 demonstrating the highest anti-inflammatory effect, both emulgel formulations significantly reduced edema compared to the untreated control group, indicating their therapeutic potential as natural alternatives for inflammatory skin conditions. The results suggest that incorporating marine-derived and plant-based oils in topical formulations could offer a promising approach to managing localized inflammation with fewer side effects than conventional synthetic agents.

### 2.4. Evaluation of the of the Healing Effect of Emulgels

The results of the wound healing effect of the tested treatments, including Gel 1 (shark liver oil emulgel), Gel 2 (shark liver oil and borage oil emulgel), and Cicatrizin^®^ (a commercial cicatrizing ointment), are presented in Table 10. These results show the evolution of the wound area over a period of 12 days, measured in mm^2^, with values reported as the average ± standard deviation (SD).

Both emulgels (Gel 1 and Gel 2) demonstrated a significant reduction in wound area over the 12-day period, with Gel 2 showing a slightly more pronounced effect than Gel 1.

The untreated control group showed a steady reduction in wound area over time, but at a slower rate than the treated groups. By day 12, the wound area reached 36.42 ± 0.13 mm^2^, indicating some degree of healing but still significant wound presence.

Cicatrizin^®^ reduced the wound area to 16.81 ± 0.27 mm^2^ by day 12, whereas both formulations (Gel 1 and Gel 2) achieved complete wound healing within the same period (wound area = 0 mm^2^). This suggests that the commercial product exhibited a slower healing rate compared to Gel 1 and Gel 2, as shown in Table 10, which presents the progression of average wound surface values in the studied groups. Statistically, Cicatrizin^®^ significantly differed in wound healing compared to the control group on days 6, 8, 10, and 12 (*p* < 0.05 and *p* < 0.01).

Gel 1 also showed a good healing effect, with the wound area reducing to 0 mm^2^ by day 12, indicating complete healing. Statistically significant differences were observed between Gel 1 and the control group from days 4 to 12 (*p* < 0.05), with the healing effect being more pronounced over time.

Gel 2 (shark liver oil and borage oil emulgel): Similar to Gel 1, Gel 2 exhibited significant wound healing, with a complete wound closure by day 12 (0 mm^2^). Statistically significant differences were observed between Gel 2 and the control group from days 4 to 12, with a particularly strong effect on day 6 (*p* < 0.01) and day 8 (*p* < 0.05).

The ANOVA results show that the differences in wound healing between the groups were statistically significant on days 6, 8, and 10 (*p* < 0.05). Gel 1 and Gel 2 showed better healing effects than the control group. However, there were no significant differences between Gel 1 and Gel 2, suggesting that both emulgels performed similarly regarding wound healing.

The results indicate that both Gel 1 (shark liver oil emulgel) and Gel 2 (shark liver oil and borage oil emulgel) demonstrated good wound healing properties, with Gel 2 showing a slight edge in the early stages of healing. Both formulations were comparable in their efficacy, and both performed better than the untreated control group. Cicatrizin^®^ outperformed the emulgels regarding wound healing at later time points, highlighting its stronger therapeutic potential.

**Figure 11:**
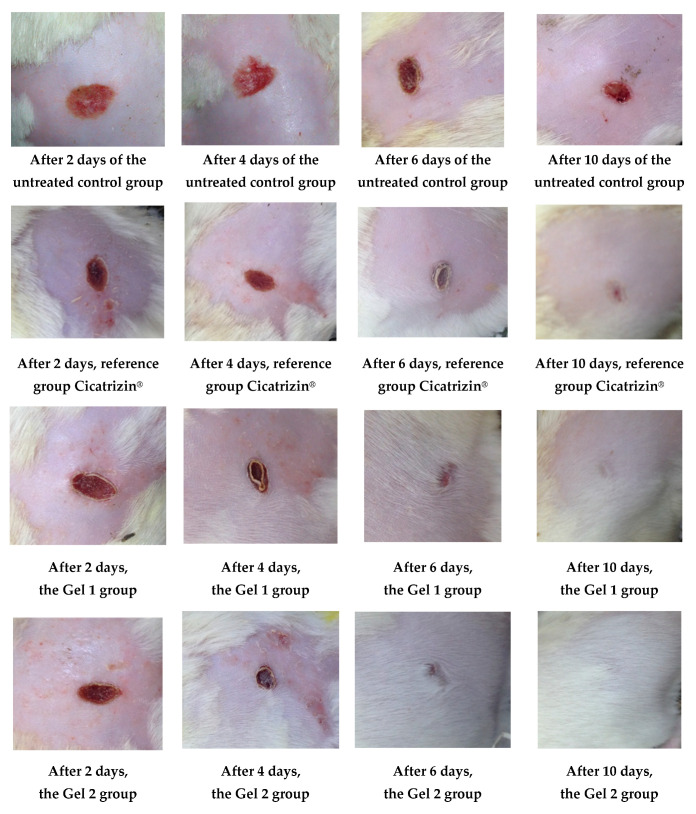
The evolution of the wounds at different times.

Figure 11 illustrates the wound healing process over time, highlighting the superior healing rates observed in groups treated with emulgels, particularly Gel 2, which contains both shark liver oil and borage oil. The progression of wound healing was significantly faster in these treated groups compared to the untreated control group, with Gel 2 demonstrating the most pronounced effects.

Gel 2 exhibited notably accelerated healing compared to Gel 1 (shark liver oil emulgel), especially in the early stages (as evidenced by the progressive reduction in wound surface area over time according data from Table 10), where it facilitated faster restoration of the depilated area (hair regrowth). This enhanced effect is attributed to the synergistic action of shark liver oil, rich in omega-3 fatty acids with anti-inflammatory and regenerative properties, and borage oil, which contains GLA, known for its potent tissue repair and anti-inflammatory benefits.

While Gel 1 also promoted wound healing, its effects were less pronounced due to the absence of borage oil. Restoring the depilated area, a key indicator of effective skin regeneration, was more efficient in Gel 2-treated animals, suggesting a more comprehensive healing process, including hair follicle regeneration.

The data from Figure 11 and Table 10 support the conclusion that combining shark liver oil and borage oil in Gel 2 enhances wound healing and accelerates tissue regeneration more effectively than shark liver oil alone. This dual-action formulation presents a promising therapeutic option for wound healing, offering a synergistic effect superior to single-oil treatments in clinical applications.

**Figure 12:**
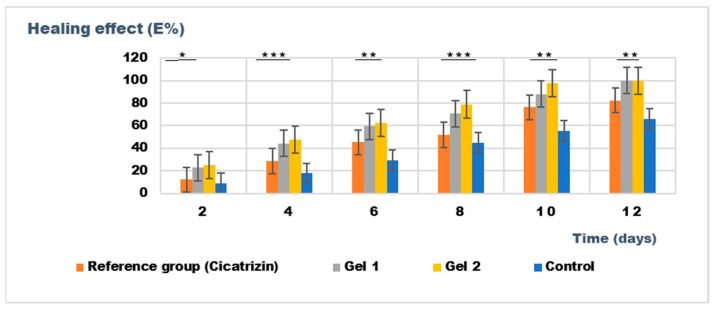
The evolution of the healing effect (E%) during the treatment. * *p* < 0.05, ** *p* < 0.01, and *** *p* < 0.001 refer to statistical significance between the reference group and tested group: Gel 1—emulgel with shark liver oil; Gel 2—emulgel with shark liver oil and borage oil; control—untreated control group.

The results presented in Figure 12 highlight the superior wound healing potential of the emulgel formulation incorporating both shark liver oil and borage oil (Gel 2).

Combining these two oils in Gel 2 appears to have a complementary effect, improving wound contraction rates and leading to faster restoration of the hair-depilated area, suggesting improved dermal regeneration. Compared to the reference pharmaceutical preparation, Cicatrizin^®^, which also showed significant wound healing effects, Gel 2 exhibited superior performance, indicating that the emulgel formulation based on marine-derived and botanical lipids provides an efficient alternative to conventional cicatrizing agents. The findings support the potential of combining natural lipid-based bioactives in dermatocosmetic formulations to enhance skin repair while maintaining an eco-friendly and biocompatible profile.

Pharmaceutical interest in species like *Squalus acanthias* usually focuses on their unique biochemical properties, including those found in their immune systems, oils, or other natural compounds that might have therapeutic applications (e.g., anticancer, anti-inflammatory effects).

There is a growing demand for sustainable alternatives to the direct harvesting of wild populations. Among these approaches, biotechnology enables the laboratory synthesis of target compounds, while synthetic biology facilitates the engineering of microorganisms to biosynthesize biologically active molecules naturally occurring in these species. Breeding *Squalus acanthias* in hatcheries is theoretically possible but practically difficult. These species are slow-growing and have low reproductive rates, complicating large-scale breeding programs. Additionally, their specific environmental requirements make controlled breeding conditions a challenge. However, some aquaculture and marine biology research efforts have focused on breeding and raising shark species in captivity, though it is less common for species like *Squalus acanthias* [70]. These efforts often aim to provide a sustainable source of shark products and reduce the impact on wild populations. Promoting sustainable practices and developing alternatives like lab-based synthesis or aquaculture could help mitigate the negative impact on these species and support conservation efforts.

## 3. Conclusions

The current study presents an original approach to the eco-friendly extraction and formulation of Black Sea shark liver oil-based emulgel, enhanced with borage oil, for dermatocosmetic applications, specifically targeting anti-inflammatory and wound healing properties. The formulations, Gel 1 (shark liver oil-based) and Gel 2 (shark liver oil combined with borage oil), demonstrated excellent rheological stability, suitable spreadability, and consistency, making them viable candidates for topical applications. Both emulgel formulations exhibited significant anti-inflammatory effects, effectively inhibiting edema induced by kaolin and dextran. Gel 2 showed superior performance, particularly in terms of faster wound healing and efficient skin regeneration. This study highlights the potential of combining shark liver oil with borage oil, rich in omega-3 and omega-6 fatty acids, to produce an eco-friendly emulgel with dual anti-inflammatory and healing actions. The research introduces a sustainable formulation that could offer new possibilities for dermatological treatments, providing a promising alternative to synthetic anti-inflammatory and healing agents in the cosmetic and pharmaceutical industries.

## 4. Materials and Methods

### 4.1. Extraction of Black Sea Shark (Squalus acanthias) Liver Oil

The laboratory extraction process for Black Sea shark (*Squalus acanthias*) liver oil was designed to obtain a high-quality oil, free from toxic solvent residues and without exposure to high temperatures that could degrade thermolabile bioactive compounds, such as polyunsaturated fatty acids (PUFAs) and fat-soluble vitamins (e.g., vitamins A, D, and E).

Livers from specimens of *Squalus acanthias* caught in the Black Sea in August 2024 were used for oil extraction. Immediately after collection, the livers were thoroughly washed with cold distilled water to remove blood and surface contaminants. The cleaned livers were then rapidly frozen at −20 °C to prevent enzymatic degradation and lipid oxidation.

Once frozen, the livers were minced using a stainless-steel grinder under controlled conditions to prevent excessive oxidation. The minced liver was then subjected to controlled thermal processing in a water bath at 50–55 °C for 30–45 min. This temperature range was carefully maintained to facilitate oil release while minimizing the thermal degradation of bioactive compounds.

**Figure 13:**
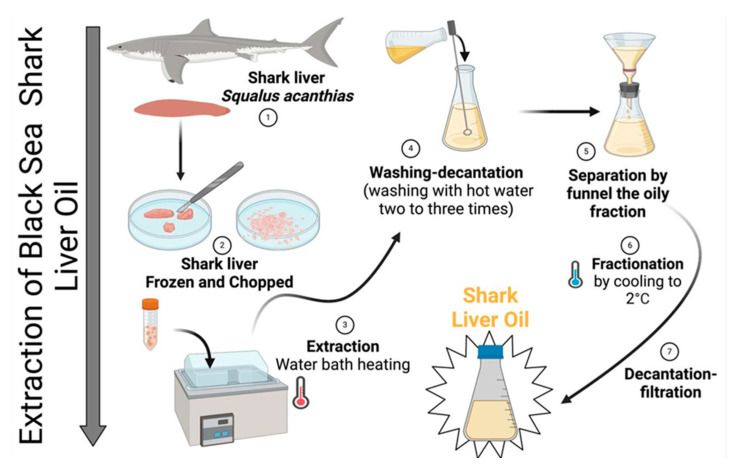
Technological process of obtaining shark (*Squalus acanthias*) liver oil.

The crude oil fraction was separated from solid residues via filtration through a fine mesh filter. The extracted oil was washed 2–3 times with warm distilled water (40–45 °C) in a separatory funnel to remove residual proteins, phospholipids, and other hydrophilic impurities. After washing, the oil was subjected to low-temperature fractionation by cooling to 2 °C for 12–24 h, allowing the saturated triglyceride fraction to crystallize and precipitate. The liquid fraction, rich in unsaturated fatty acids, was carefully separated by decantation and filtered through a 0.45 µm membrane filter to remove any remaining particulates. The purified oil was stored in dark glass containers to prevent oxidative degradation. The final product was maintained at 8–15 °C until further analysis (Figure 13).

### 4.2. Analysis of Black Sea Shark (Squalus acanthias) Liver Oil

The extracted *Squalus acanthias* liver oil was subjected to a series of physicochemical analyses to assess its quality and stability. The following parameters were determined at 20 °C in accordance with the methods described in the Romanian Pharmacopoeia, Edition X [71]: density (g/cm^3^), refractive index, acid value (mg KOH/g oil), iodine value (g I_2_/100 g oil), saponification value (mg KOH/g oil), and peroxide value (meq O_2_/kg oil).

Borage extra virgin oil, obtained from *Borage officinalis* seeds by cold pressing, was also analyzed. The oil was supplied by MAYAM and is Ecocert Greenlife certified in accordance with the COSMOS standard. The oil was purchased for the development of a dermatocosmetic formula in combination with shark liver oil.

### 4.3. Fatty Acid Composition Analysis

The fatty acid profile of *Squalus acanthias* liver oil and borage oil were determined by gas chromatography (GC) after derivatization to fatty acid methyl esters (FAMEs). An internal standard (C23:0 methyl ester; Nu-Chek Prep Inc., Elysian, MN, USA) was added to a known quantity of oil for accurate quantification. The sample was dried under a nitrogen atmosphere to remove any residual solvents.

Esterification process: hydrolysis was performed using a 7.9% potassium hydroxide (KOH) solution in methanol, breaking triglycerides into free fatty acids. After cooling, the reaction mixture was treated with a 20% boron trifluoride (BF_3_) solution in methanol to catalyze the methylation process, converting free fatty acids into their corresponding methyl esters.

Gas Chromatography (GC) Conditions:

Instrument: Shimadzu GC-17A (Kyoto, Japan)

Detector: Flame Ionization Detector (FID); column: Capillary column suitable for FAME analysis (SP-2560, 100 m × 0.25 mm i.d., 0.2 µm film thickness); carrier gas: Helium (99.999% purity) at a constant flow rate.

The chromatographic peaks were identified and quantified using a standard FAME mix (Nu-Chek Prep Inc., Elysian, MN, USA) as a reference. Peak integration and data processing were carried out using Shimadzu Class GC-10 software version 1.3.

Calibration curve equation: y = 0.0325x + 0.001; R^2^ = 0.9977

Limit of Detection (LOD) = 0.05 mg/mL; Limit of Quantification (LOQ) = 0.15 mg/mL

All analyses were performed in triplicate, and results were expressed as means ± standard deviation (SD). Analytical-grade reagents (Sigma-Aldrich, Darmstadt, Germany) were used throughout the process to ensure precision and reproducibility.

### 4.4. Formulation and Characterization of Emulgels with Shark Liver Oil

Two emulgels were prepared: one containing 5% shark liver oil emulsified in a 2% Carbopol 940 base with Tween 80 as the emulsifier and the other containing 2.5% shark liver oil and 2.5% borage oil emulsified in a 2% Carbopol 940 base (Table 11).

The preparation of the emulgels began by first preparing the base. 2% Carbopol 940 (Sigma-Aldrich, Darmstadt, Germany) was dispersed into distilled water and glycerin (purity over 99%; Glycerin from Merck, Darmstadt, Germany), and triethanolamine (TEA; purity over 99%; Triethanolamine from Carl Roth GmbH + Co. KG., Karlsruhe, Germany) was slowly added to neutralize the Carbopol, forming a gel. The mixture was allowed to rest for 24 h to ensure proper gel formation and stability. Following this period, the emulsification process commenced with the preparation of the oil phase. In the first emulgel, 5% shark liver oil was emulsified with Tween 80, whereas the second emulgel contained a blend of 2.5% shark liver oil and 2.5% borage oil, also emulsified using Tween 80. The oil phase was then gradually incorporated into the pre-hydrated Carbopol gel under continuous stirring (2000 rpm for 10 min) using a turbine stirrer (Heidolph RZR 2020, Heidolph Instruments GmbH & Co. KG, Schwabach, Germany), ensuring the formation of stable and homogeneous emulgels.

The emulgels were subjected to additional analyses for characterization, including appearance analysis, pH determination, thermal stability, viscosity, and spreadability assessment. The appearance was evaluated by examining a sample of the emulgel spread in a thin layer on a microscope slide using a magnifying glass (4.5×). To determine the pH, samples were processed by extracting with distilled water in a 1:5 ratio, then heating in a water bath at 60 °C and homogenizing for 10 min. The pH of the aqueous phase was measured using a Radelkis pH meter (Budapest, Hungary). Thermal stability was assessed by storing the samples at 2 °C and 40 °C for 8 h. A 5 g sample of the emulgel was placed in weighing vials with lids, which were stored in an oven and a refrigerator at the specified temperatures. Afterward, the appearance of the samples was examined to ensure they remained homogeneous. Viscosity was determined using a VEVOR NDJ-9S Digital Rotary Viscometer (Kansas City, MO, USA). The spreadability of the emulgel was evaluated 28 h after preparation by measuring the spreading diameter of 1 g of the sample placed between two 20 × 20 cm glass plates. The top plate was standardized at 125 g. The spreading area was measured after 1 min intervals with increasing applied weights (50 g, 100 g, 150 g, 250 g, 500 g, 750 g) in millimeters. These tests were repeated 30 days after preparation to assess any changes. Results were expressed as the spreading area as a function of the applied mass, according to the following equation:

S_i_ = d_i_^2^ (π/4)

where:

- S_i_ is the spreading area (mm^2^) resulting from the applied mass “i” (g), and
- d_i_ is the mean diameter (mm) reached by the sample.

The rheological characteristics of the emulgel samples were evaluated using a Multi-Visc Rheometer rotational viscometer (Fungilab, Mumbai, India) by conducting stationary shear analysis at a temperature of 33 °C ± 0.1 °C, which approximates human skin temperature. Temperature control during the analysis was maintained using a ThermoHaake P5 Ultrathermostat (Houston, TX, USA). Three emulgel samples were subjected to shear forces using a TR 10 standard spindle, with the shear rate varying from 0.08 to 16.8 s^−1^, corresponding to rotational speeds between 0.3 and 60 rpm. The resulting shear stress data as a function of shear rate were used to generate upward rheograms. The rheological behavior of the samples was further analyzed by applying different models that describe the relationship between shear stress (τ) and shear rate ($\dot{\gamma }$), specifically the Casson model (Equation (1)) and the Herschel–Bulkley model (Equation (2)). These models provide insight into the flow behavior and viscoelastic properties of the emulgel formulations [71,72].

(1) $$\tau^{0.5}={\tau_{0}}^{0.5}+\eta^{0.5}\cdot {\dot{\gamma }}^{0.5}$$

(2) $$\tau =\tau_{0}+K\cdot {\dot{\gamma }}^{n}$$

where η is the plastic viscosity (Pa·s), τ_0_ is the yield stress (Pa), K is the consistency index (Pa·s^n^), and n is the flow index (dimensionless). The flow parameters were determined using Table Curve 2D software version 5.01.

For internal morphology analysis, the emulgel samples were examined using a Scanning Electron Microscope (SEM) (Thermo Fisher Scientific, GmbH, Dreieich, Germany). SEM imaging was performed by scanning the samples with an electron beam at an accelerating voltage of 15 kV and a magnification range of 2400 to 2500×. This technique provided detailed visual insights into the structural characteristics and surface morphology of the emulgel formulations.

### 4.5. Evaluation of the Anti-Inflammatory Activity of Emulgels

For this experiment, six groups of 10 male Wistar rats weighing 230 ± 15 g were used. The animals were kept in laboratory conditions for 2 days to acclimate to their new environment (experimental room temperature 22 ± 2 °C, humidity 40–50%). The diet consisted of feeding at 8:00 and 17:00, with water ad libitum. All animal procedures were approved by the Scientific Research Ethics Commission of Carol Davila University of Medicine and Pharmacy in compliance with the Animal Protection Act (code of ethical conduct 6485/12.03.2024).

Acute inflammation was induced using two experimental methods: kaolin-induced edema and dextran-induced edema. Kaolin stimulates the formation of prostaglandins, causing local inflammation and edema, while dextran induces edema mainly through the release of histamine and serotonin, resulting in anaphylactoid edema. Edema was induced by intra-plantar injection of 0.1 mL of 10% kaolin suspension and 0.2 mL of 6% dextran solution.

For each edematous agent, three groups of 10 male Wistar rats were used:

Group 1: Control group (untreated);

Group 2: Reference group treated with diclofenac gel 10 mg/g (Fiterman company);

Group 3: Test group treated with emulgel containing shark liver oil (Gel 1);

Group 4: Test group treated with emulgel containing shark liver oil and borage oil (Gel 2).

The anti-inflammatory effects of the emulgels were evaluated by comparing the volume changes in the paw treated with the tested preparations against the control group and the Diclofenac gel. All animals received the edematous agent, and the test preparation was applied in a thin layer (~0.25 g) on the paw where edema was induced.

The volume of the rat’s paw was measured using a plethysmometer Ugo Basile 7140 (Gemonio, Italy) at multiple intervals: 2 h, 4 h, 6 h, and 24 h after kaolin injection, and 30 min, 60 min, 90 min, and 120 min after dextran injection. The average anti-inflammatory effect (in mL) and the percentage of edema inhibition for each group were calculated using the following formula:

Edema inhibition effect (I) % = (X control − X treatment agent/X control) × 100

where X treatment agent represents the average value of the edema produced by the tested gel (diclofenac gel or emulgels), and X control represents the average value of the edema produced in the control group in the same time interval after administering the edematous agent.

### 4.6. Evaluation of the Healing Activity of Emulgels

For this experiment, four groups of 10 male Wistar rats weighing 200 ± 10 g were used. The rats were acclimatized in laboratory conditions for 2 days (experimental room temperature 22 ± 2 °C, humidity 40–50%). The animals were fed twice daily at 8:00 and 17:00, with water ad libitum in bottles. All animal procedures were approved by the Scientific Research Ethics Commission of Carol Davila University of Medicine and Pharmacy under the Animal Protection Act (code of ethical conduct 6485/12.03.2024).

Wounds were induced on the shaved backs of the animals by ether anesthesia and applying a heated metal disk (1 cm in diameter), which was maintained for 10 s on the shaved area after being heated in water with 5% NaCl at 105 °C. The animals were then randomly assigned to the following groups:

Group 1: Control group (untreated);

Group 2: Reference group treated with Cicatrizin^®^ (Tis Farmaceutic S.A., Bucharest, Romania), a cicatrizing ointment containing natural plant extracts (mallow, St. John’s wort, chamomile, and calendula);

Group 3: Test group treated with emulgel containing shark liver oil;

Group 4: Test group treated with emulgel containing shark liver oil and borage oil.

The treatment was applied twice a day for 12 days, and wound healing was observed by measuring the treated areas (in mm^2^) every two days. The healing effects were compared between the different groups. The clinical condition of the animals was monitored throughout the study.

The healing effect (E%) was calculated using the following formula:

E % = (A_i_ − A_i+1_/A_i_) × 100

where A_i_ is the average wound surface at the initial moment (i), and A_i+1_ is the average wound surface at the moment i + 1.

### 4.7. Statistical Analysis

All determinations were performed in triplicate to ensure accuracy and reproducibility, and the results were expressed as mean ± standard deviation (SD). The mean provides a central value for the data, while the SD quantifies the variation or spread of the data around the mean. Statistical analysis was carried out using Student’s *t*-test and analysis of variance (ANOVA) to assess the significance of the differences between the treatment and control groups.

The Student’s *t*-test was used to compare the two groups, specifically to analyze the differences between the control group and a single treatment group (such as the emulgel with shark liver oil or shark liver oil and borage oil). The *t*-test assesses whether the means of the two groups are statistically different from each other.

Analysis of Variance (ANOVA) was used when comparing multiple groups simultaneously, such as the control group, Cicatrizin^®^-treated group, shark liver oil emulgel group, and shark liver oil and borage oil emulgel group. ANOVA tests the hypothesis that there are no significant differences between the means of the groups. If ANOVA showed significant differences, post-hoc tests (such as Tukey’s test) were used to identify which specific groups differed [73,74].

A *p*-value < 0.05 was considered statistically significant for all statistical tests, indicating that the observed differences were unlikely to have occurred by chance. All analyses were performed using statistical software, and the results were interpreted based on the confidence intervals and *p*-values derived from the tests.

For the linearity of rheological properties, the data were subjected to a regression analysis to assess the relationship between shear stress (τ) and shear rate ($\dot{\gamma }$) across the range of applied shear rates. The linearity of the rheological behavior of the emulgel formulations (shark liver oil and shark liver oil with borage oil) was analyzed by plotting shear stress versus shear rate and examining the correlation between the two parameters.

To evaluate the linearity, the data were fitted to different rheological models, such as the Newtonian model for simple systems and the Herschel–Bulkley model or Casson model for more complex, non-Newtonian behavior, depending on the observed flow characteristics of the samples.

A linear regression analysis was performed for the datasets obtained at low shear rates, where the system behaves more predictably. The correlation coefficient (R^2^) was calculated to quantify the strength of the linear relationship between shear stress and shear rate. A high R^2^ value (typically R^2^ > 0.98) indicates that the data fit well to a linear model, implying Newtonian behavior in this range. If the R^2^ value was lower, non-Newtonian behavior might have been observed, requiring the use of more complex models (like Herschel–Bulkley or Casson).

## Figures and Tables

**Table 1:** Values of examined parameters relative to the oil samples (average value ± SD).

Parameter	Shark Liver Oil	Borage Oil
Iodine value (g I_2_/100 g fatty acids)	163.22 ± 0.66	152.36 ± 0.33
Acid value (mg KOH/g sample)	3.93 ± 0.33	3.68 ± 0.55
Saponification value (mg KOH/g sample)	185.07 ± 0.25	192.18 ± 0.18
Peroxide index (mEq O_2_/kg)	2.65 ± 0.55	3.84 ± 0.65
Density at 20 °C (g/cm^3^)	0.94 ± 0.36	0.92 ± 0.25
Refractive index at 20 °C	1.48 ± 0.57	1.48 ± 0.42

SD—standard deviation.

**Table 2:** Percent distribution of fatty acids in the oil samples.

Fatty Acid	Shark Liver Oil mg/g ± SD (%)	Borage Oil mg/g ± SD (%)
C 10: 0	0.12 ± 0.23	0.36 ± 0.41
C 12: 0	0.46 ± 0.13	0.82 ± 0.26
C 14: 0	1.23 ± 0.45	0.93 ± 0.44
C 15: 0	0.46 ± 0.16	ND
C 16: 0	14.15 ± 1.84	15.87 ± 1.22
C 17: 0	0.82 ± 0.28	ND
C 18: 0	5.75 ± 0.33	5.96 ± 0.65
C 20: 0	0.87 ± 0.18	0.48 ± 0.14
C 22: 0	0.41 ± 0.24	0.39 ± 0.34
C 24: 0	0.26 ± 0.55	0.41 ± 0.52
C 26: 0	0.08 ± 0.35	ND
C 14: 1	0.11 ± 0.33	0.31 ± 0.19
C 16: 1	4.23 ± 0.52	0.82 ± 0.11
C 16: 1ω-7	2.92 ± 0.81	ND
C 17: 1	0.27 ± 0.18	ND
C 18: 1	7.72 ± 0.73	13.37 ± 0.28
C 18: 1ω-7	3.75 ± 0.33	ND
C 18: 1ω-9	2.81 ± 0.74	ND
C 20: 1	2.19 ± 0.38	0.94 ± 0.63
C 22:1	0.37 ± 0.14	0.47 ± 0.11
C 24:1	0.41 ± 0.12	0.36 ± 0.34
C 18: 2ω-6	4.48 ± 0.52	29.86 ± 1.16
C 18: 3	0.25 ± 0.25	ND
C 18: 3ω-6	1.92 ± 0.14	26.66 ± 0.37
C 20:3	2.48 ± 0.24	0.40 ± 0.22
C 20: 4ω-6	1.68 ± 0.55	ND
C 20: 5ω-3	16.68 ± 0.28	0.72 ± 0.51
C 22: 5ω-3	2.69 ± 0.16	ND
C 22: 6ω-3	18.14 ± 0.31	0.87 ± 0.14
C 20: 2ω-6	0.82 ± 0.44	ND
C 18: 4ω-3	1.38 ± 0.62	ND
Σ ω-3	38.89	1.59
Σ ω-6	8.9	56.52
ω-3/ω-6	4.36	0.03
Polyunsaturated fatty acids/Saturated fatty acids	2.44	2.3
Polyunsaturated fatty acids/Monounsaturated fatty acids	3.92	3.48

SD—standard deviation; ND—not detected.

**Table 3:** Characteristics of emulgels.

Characteristics	Gel 1	Gel 2
Initial organoleptic evaluation	appearance: homogeneous; color: yellowish; smell: specific	appearance: homogeneous; color: yellow; smell: specific
Organoleptic evaluation after 30 days	maintenance of the original characteristics unchanged	maintenance of the original characteristics unchanged
Organoleptic evaluation after 60 days	maintenance of the original characteristics unchanged	maintenance of the original characteristics unchanged
pH—initial	4.7–5.0	4.9–5.2
pH—after 30 days	4.7–5.0	4.9–5.2
pH—after 60 days	5–5.3	5.2–5.5
Viscosity—initial	881.92 ± 1.31 Pa·s	843.88 ± 2.33 Pa·s
Viscosity—after 30 days after 30 days	875.87 ± 2.63 Pa·s	831.96 ± 1.72 Pa·s
Viscosity—after 60 days	857.97 ± 1.42 Pa·s	801.89 ± 1.24 Pa·s
Thermal stability initial	good stability without a tendency to separate	good stability without a tendency to separate
Thermal stability after 30 days	good stability without a tendency to separate	good stability without a tendency to separate
Thermal stability after 60 days	good stability without a tendency to separate	good stability without a tendency to separate

**Table 4:** Herschel–Bulkley model flow parameters obtained through stationary shear analysis at 33 °C.

Gel/Flow Parameters	Yield Stress (τ_0_) (Pa)	Consistency Index (K) (Pa⋅s^n^)	Flow Index (n)	Viscosity at 0.3 rpm (η_0.3_) (Pa⋅s) Initial	Viscosity at 0.3 rpm (η_0.3_) (Pa⋅s) After 30 Days
**Gel 1**	46.233	32.738	0.290	882.700	876.200
**Gel 2**	52.366	34.114	0.280	844.600	832.600

τ_0_—minimum shear stress required to initiate flow in gel sample.

**Table 5:** The coefficient values of rheological models tested at 33 °C.

Emulgel/Rheological Model	Casson	Herschel–Bulkley
Gel 1	0.856	0.988
Gel 2	0.944	0.992

**Table 6:** Parameters of the Ostwald–de Waele model for shark liver oil.

Parameter	K-Consistency Index	n-Flow Index	R-Correlation Coefficient
Test 1	9.453	0.476	0.995
Test 2	9.117	0.443	0.996
Test 3	9.721	0.501	0.995
Average	9.430	0.473	-
DS	0.303	0.029	-
CV(%)	3.209	6.143	-

**Table 7:** Parameters of the Ostwald–de Waele model for shark liver oil and borage oil emulgel.

Parameter	K-Consistency Index	n-Flow Index	R-Correlation Coefficient
Test 1	9.221	0.451	0.998
Test 2	9.665	0.551	0.999
Test 3	8.912	0.392	0.998
Average	9.266	0.465	-
DS	0.379	0.080	-
CV(%)	4.086	17.298	-

**Table 8:** The anti-inflammatory effect of emulgels on inflammatory edema induced with 10% kaolin suspension.

Group	Edema 2 h (mL) $(\bar{X}$ ± SD)	Edema 4 h (mL) $(\bar{X}$ ± SD)	Edema 6 h (mL) $(\bar{X}$ ± SD)	Edema 24 h (mL) $(\bar{X}$ ± SD)
Control group	0.228 ± 0.031 **	0.274 ± 0.055 **	0.340 ± 0.052 *	0.293 ± 0.036 *
Tested group (Gel 1)	0.148 ± 0.042 *	0.146 ± 0.061 *	0.156 ± 0.012 *	0.106 ± 0.025 **
Tested group (Gel 2)	0.140 ± 0.012 *	0.138 ± 0.018 *	0.142 ± 0.028 *	0.098 ± 0.016 **
Reference group (Diclofenac gel)	0.128 ± 0.041 *	0.120 ± 0.043 *	0.112 ± 0.028 *	0.085 ± 0.046 *
F (ANOVA)	1.648	3.736	1.074	4.162
P (ANOVA)	0.434	0.822	0.072	0.246

$\bar{X}$ ± SD = average value ± standard deviation; * *p* < 0.05, ** *p* < 0.01.

**Table 9:** The anti-inflammatory effect of emulgels on inflammatory edema induced with dextran solution 6%.

Group	Edema 30 min. (mL) $(\bar{X}$ ± SD)	Edema 60 min. (mL) $(\bar{X}$ ± SD)	Edema 90 min. (mL) $(\bar{X}$ ± SD)	Edema 120 min. (mL) $(\bar{X}$ ± SD)
Control group	0.208 ± 0.025 **	0.226 ± 0.052 **	0.254 ± 0.033 *	0.266 ± 0.055 **
Tested group (Gel 1)	0.132 ± 0.041 *	0.136 ± 0.011 *	0.132 ± 0.046 **	0.128 ± 0.021 *
Tested group (Gel 2)	0.126 ± 0.014 *	0.122 ± 0.011 *	0.120 ± 0.016 **	0.114 ± 0.034 *
Reference group (Diclofenac gel)	0.112 ± 0.034 *	0.104 ± 0.036 *	0.098 ± 0.022 *	0.080 ± 0.044 **
F (ANOVA)	3.056	1.624	5.066	1.284
P (ANOVA)	0.608	0.418	0.224	0.104

$\bar{X}$ ± SD = average value ± standard deviation; * *p* < 0.05, ** *p* < 0.01.

**Table 10:** Evolution of the wound healing effect: average wound area (mm^2^) ± SD (standard deviation).

Group	Initial	2 Days	4 Days	6 Days	8 Days	10 Days	12 Days
Control	108.02 ± 0.25 *	98.22 ± 0.21 *	88.81 ± 0.11 *	76.41 ± 0.33 *	59.61 ± 0.19 **	48.21 ± 0.32 *	36.42 ± 0.13 *
Cicatrizin^®^	96.02 ± 0.33 *	84.21 ± 0.14 *	68.61 ± 0.75 **	52.42 ± 0.21 **	46.22 ± 0.85 *	22.81 ± 0.74 *	16.81 ± 0.27 *
Gel 1	104.41 ± 0.25 *	80.61 ± 0.65 *	58.22 ± 0.36 *	42.21 ± 0.24 *	30.62 ± 0.28 *	12.42 ± 0.14 *	0
Gel 2	104.02 ± 0.66 *	78.22 ± 0.55 **	54.61 ± 0.15 *	38.82 ± 0.64 *	21.81 ± 0.12 *	2.41 ± 0.48 *	0
F (ANOVA)	0.846	3.034	0.621	5.286	1.664	1.416	1.314
P (ANOVA)	0.442	0.068	0.056	0.014	0.208	0.254	0.302

* *p* < 0.05, ** *p* < 0.01.

**Table 11:** Emulgels composition.

Components	Gel 1	Gel 2
Carbopol 940	2.0 g	2.0 g
Glycerin	5.0 g	5.0 g
Triethanolamine	q.s.	q.s.
Tween 80	0.5 g	0.5 g
Shark liver oil	5.0 g	2.5 g
Borage oil	-	2.5 g
Purified water	until 100 g	until 100 g

q.s. = quantum satis.

## Data Availability

The original contributions presented in the study are included in the article; further inquiries can be directed to the corresponding authors.
